# Antioxidant Activity and Phenolic Composition of Amaranth (*Amaranthus caudatus*) during Plant Growth

**DOI:** 10.3390/antiox8060173

**Published:** 2019-06-12

**Authors:** Magdalena Karamać, Francesco Gai, Erica Longato, Giorgia Meineri, Michał A. Janiak, Ryszard Amarowicz, Pier Giorgio Peiretti

**Affiliations:** 1 Institute of Animal Reproduction and Food Research, Polish Academy of Sciences, Tuwima 10, 10-748 Olsztyn, Poland; m.janiak@pan.olsztyn.pl (M.A.J.); r.amarowicz@pan.olsztyn.pl (R.A.); 2 Institute of Sciences of Food Production, National Research Council, 10095 Grugliasco, Italy; francesco.gai@ispa.cnr.it (F.G.); piergiorgio.peiretti@ispa.cnr.it (P.G.P.); 3 Department of Veterinary Sciences, University of Turin, 10095 Grugliasco, Italy; erica.longato@unito.it (E.L.); giorgia.meineri@unito.it (G.M.)

**Keywords:** amaranth, morphological stage, scavenging activity, ferrous ions chelating ability, reducing power, phenolic compounds, rutin, growth cycle

## Abstract

The antioxidant activity and phenolic composition of the aerial part of *Amaranthus caudatus* at seven stages of development were investigated. Total phenolic content, ABTS^•+^, DPPH^•^, and O_2_^•−^ scavenging activity, ferric-reducing antioxidant power (FRAP), and Fe^2+^ chelating ability were evaluated. The phenolic profile was characterized by 17 compounds. Rutin was predominant in all growth stages, although its content, similar to the quantity of other phenolics, changed during the growth cycle. Flavonols were most abundant in the plants of early flowering and grain fill stages. In contrast, the highest content of hydroxycinnamic acid derivatives was found in the early vegetative stage. The results of antioxidant assays also showed significant differences among plant stages. Generally, the lowest antioxidant activity was found in the shooting and budding stages. Significantly higher activity was observed in amaranths in earlier (vegetative) and later (early flowering and grain fill) stages, suggesting that plants in these stages are valuable sources of antioxidants.

## 1. Introduction

Amaranth (*Amaranthus* spp.) is a gluten-free pseudocereal that is cultivated primarily in Mexico and South America, but also thrives in all temperate-tropical areas of the world [1]. Moreover, in certain regions of the world, such as eastern Africa, amaranth leaves are consumed as a vegetable because it is a fast-growing plant available most of the year. There has been a renewed interest in this ancient and highly nutritious food crop due to the excellent nutritional value of seed and leaves [2,3,4]. Both seeds and leaves are rich sources of proteins, which constitute up to 15–43% and 14–30% of fresh matter (FM), respectively. Amaranth proteins have a well-balanced amino acid composition [4], high bioavailability [3], and good functional properties [5]. Dietary fiber, vitamins and precursors of vitamins (ascorbic acid, riboflavin, tocols, carotenoids), as well as minerals (Ca, Fe, Mg, K, Cu, Zn, and Mn) are other important nutrients present in seeds and leaves of amaranth. Their contents are high compared to these in some cereals and green leafy vegetables [1,2,4]. Nutrient composition causes an increasing interest in amaranth as a food ingredient, especially in the production of gluten-free products [6,7].

In addition to macro- and micronutrients, amaranth contains secondary plant metabolites, which may play a significant role in the human diet due to their potential health beneficial effects [8]. Considerable research has been conducted over the past years on phenolic compound profile of amaranth seeds [8,9,10,11] and their functional and bioactive properties [12,13,14,15]. However, recent findings confirmed that leaves and other aerial parts of *Amaranthus* also are important sources of phenolic compounds [16,17,18]. Among phenolic compounds, those belonging to hydroxycinnamic acids, benzoic acids, and flavonols and their glycosides were identified in amaranth leaves, flowers, and stalks [18,19]. Steffensen et al. [20] found additional hydroxycinnamyl amides in aerial parts of young plants. Phenolic compounds are well-known as antioxidants. Conforti et al. [21] showed that amaranth leaf extracts contained phenolics, inhibited nitric oxide production, and scavenged free radicals. The reducing potential and antioxidant activity in lipid systems of various parts of amaranth shoot system were determined as well [16,19,22]. Other phytochemicals with antioxidant activity which occur in amaranth are betalains, especially betacyanins [23]. The contents of these pigments vary among *Amaranthus* species and genotypes [24]. If they are present in the plant, they are accumulated mainly in seedlings, leaves, and inflorescences.

Although the phenolic compound and betacyanin profiles of individual botanical parts of amaranth as well as their antioxidant potential are well-known, information about effects of the growth cycle on these phytochemicals and their activity is still scarce. It is important to identify the changes in antioxidant activity and phenolic compound composition by the growth stage of the amaranth.

In a previous paper [25], we reported the nutritive characteristics of *A. caudatus*, and in particular, we evaluated the effects of plant aging on the chemical composition, gross energy, *in vitro* true digestibility, neutral detergent fiber digestibility and fatty acid profile. However, to the best of our knowledge, no research has been conducted to investigate the change of antioxidant activities and phenolic composition of extracts of *A. caudatus* during plant growth. Barba de la Rosa et al. [26] stated that the knowledge of *Amaranthus* spp. as a source of phytochemicals will increase their importance as a potential source of antioxidant compounds in the human diet.

The aim of this study was to compare the antioxidant activity, TPC, and individual phenolic compounds of *A. caudatus* during plant growth, to identify the ideal growing stage to achieve the maximum antioxidant properties, and to optimize its use as a source of antioxidants for use in food production.

## 2. Materials and Methods 

### 2.1. Chemicals

The Folin-Ciocalteu phenol reagent (FCR), gallic acid, 2,2-diphenyl-1-picrylhydrazyl (DPPH), 2,2’-azinobis-(3-ethylbenzothiazoline-6-sulfonic acid) (ABTS), 2,4,6-tri(2-pyridyl)-s-triazine (TPTZ), 6-hydroxy-2,5,7,8-tetramethyl-chroman-2-carboxylic acid (Trolox), ferrozine, caffeic acid, quercetin, *p*-coumaric acid, ferulic acid, rutin, and kaempferol-3-*O*-rutinoside were obtained from Sigma-Aldrich (St. Louis, MO, USA). Methanol, acetonitrile, sodium carbonate, ferric chloride, ferrous chloride, ferrous sulfate, and potassium persulfate were acquired from Avantor Performance Materials (Gliwice, Poland). The ACL kit was purchased from Analytik Jena (Jena, Germany).

### 2.2. Plant Material

*A. caudatus* seeds were generously donated by Pedon S.p.A. (Molvena, Italy). The amaranth stands were seeded in the spring in field trials (Department of Veterinary Science of the University of Turin) located in Grugliasco, Piedmont, NW Italy (293 m a.s.l., 45°03’57.9”N 7°35’36.9”E), and no irrigation or fertilizers were applied after sowing. The herbage samples were collected in the morning after evaporation of dew and never collected on rainy days. Sampling was performed according to Peiretti et al. [25] at seven progressive morphological stages from early vegetative to grain fill stage from May to July 2014. Plants were cut to a 1–2 cm stubble height with edging shears from two replicate 2 m^2^ subplots randomly located in 2 × 7 m^2^ plots.

### 2.3. Extraction

Plant samples were lyophilized (FreeZone Freeze Dry System, Labconco, Kansas City, MO, USA) and ground. Three-gram portions of materials were suspended in 30 mL of 80% (*v/v*) methanol. The closed bottles containing suspensions were shaken for 15 min in a water bath (SW22, Julabo, Seelbach, Germany) at 65 °C. After filtration, the residues were extracted twice more. Combined filtrates for each sample were dried by evaporation of methanol under vacuum at 50 °C (Rotavapor R-200, Büchi Labortechnik, Flawil, Switzerland) and lyophilization of remaining water. Yield of extraction (%) was calculated on the basis of weight of plant sample and dry extract.

### 2.4. Total Phenolic Content

The colorimetric reaction with FCR was carried out to determinate the total phenolic content (TPC) [27]. The 0.25 mL of extract solution in methanol (1.25 mg/mL), 0.25 mL of FCR, 0.5 mL of saturated sodium carbonate solution, and 4 mL of water were mixed. After 25 min, the reaction mixtures were centrifuged (5 min, 5000× *g*, MPW-350R, MPW Med. Instruments, Warsaw, Poland), and the absorbance of supernatants was measured at 725 nm (DU-7500 spectrophotometer, Beckman Instruments, Fullerton, CA, USA). TPC was expressed as gallic acid equivalents (GAE) per g of extract or per g of FM of plant.

### 2.5. Trolox Equivalent Antioxidant Capacity

The Trolox equivalent antioxidant capacity (TEAC) was determined according to the assay reported by Re et al. [28]. ABTS^•+^ was generated and diluted as in the original description. Next, solutions of ABTS^•+^ (2 mL) and amaranth extracts (20 μL with a concentration of 2.5 mg/mL) were vortexed and heated at 30 °C (TH-24 block heater, Meditherm, Warsaw, Poland) within 6 min. After incubation, absorbance was measured at 734 nm (DU-7500 spectrophotometer). The results were expressed as μmol Trolox equivalents (TE) per g of extract or per g of FM of plant.

### 2.6. Ferric-Reducing Antioxidant Power

The Benzie and Strain method [29] was used to evaluate the ferric-reducing antioxidant power (FRAP) of amaranth extracts. First, FRAP reagent was prepared. Next, the portions of 2.25 mL of this reagent were mixed with 225 μL of water and 75 μL of aqueous solution of amaranth extracts (1 mg/mL). Absorbance of mixtures was recorded at 593 nm after incubation at 37 °C for 30 min. FRAP results were expressed as μmol Fe^2+^ equivalents per g of extract or per g of FM using the calibration curve for FeSO_4_.

### 2.7. Photochemiluminescence Assay

In the photochemiluminescence (PCL) assay, superoxide radical anions (O_2_^•−^) were generated from luminol [30]. The antiradical activity against these radicals was determined using the ACL (antioxidant capacity of lipid-soluble substances) kit (Analytik Jena, Jena, Germany). The amaranth extracts were dissolved in methanol to a concentration of 0.25 mg/mL. The portions of 10 μL of solutions were mixed with 2.3 mL of methanol (reagent 1), 200 μL of buffer solution (reagent 2), and 25 μL of luminol (reagent 3). The Photochem device (Analytik Jena) supported by PCLsoft software was used to perform reactions and calculate the results, which were expressed as µmol of Trolox equivalents (TE) per g of extract or per g of FM of plant.

### 2.8. Fe^2+^ Chelating Ability

The ability of amaranth extracts to chelate ferrous ions was determined by a method with ferrozine [31] that was modified to be performed with multi-well plates [32]. Extract solution in water (0.25 mg/mL), 0.4 mM FeCl_2_ × 4H_2_O and 5 mM ferrozine were mixed in a ratio of 10:1:2 (*v/v/v*). After 10 min, absorbance was measured at 562 nm using an Infinite M1000 microplate reader (Tecan, Männedorf, Switzerland). The chelating ability was expressed as percentage of Fe^2+^ bound.

### 2.9. Scavenging of the DPPH Radicals

The DPPH^•^ scavenging activity of amaranth extracts was evaluated by the method of Brand-Williams et al. [33]. Extract solutions in methanol over a range of concentrations from 2–10 mg/mL were prepared. To 100 μL of these solutions, 0.25 mL of 1 mM DPPH and 2 mL of methanol were added. After 20 min, absorbance of the mixtures was read at 517 nm. The curves of absorbance values vs. concentration of samples (mg/assay) were plotted. Additionally, EC_50_ values defined as concentration of extract (mg/mL reaction mixture) needed to scavenge the 50% of initial DPPH^•^ were evaluated.

### 2.10. Analysis of Phenolic Compounds

The HPLC-DAD Shimadzu system (Shimadzu, Kioto, Japan), which consisted of a CBM-20A controller, DGU-20A5R degassing unit, two LC-30AD pumps, SIL-30AC autosampler, SPD-M30A diode array detector (DAD), and CTO-20AC oven, was used to analyze the phenolic compound of the amaranth extracts. Extracts dissolved in 80% (*v/v*) methanol were injected (10 μL) into a Luna C8(2) (4.6 × 150 mm, particle size 3 μm, Phenomenex, Torrance, CA, USA) column [34]. The mobile phase consisted of solvents A (acetonitrile-water-trifluoroacetic acid, 5:95:0.1, *v/v/v*) and B (acetonitrile-trifluoroacetic acid, 100:0.1, *v/v*): From 0–16 min, the eluent composition was changed from 0–24% B. The flow rate was 1 mL/min. The oven temperature was 25 °C and detector wavelength ranged from 200 to 600 nm. The content of individual phenolic compounds in the extracts was expressed on the basis of a calibration curve of the corresponding standards or structurally related substances. 

For identification of extract compounds, HPLC-MS/MS analysis was carried out using a liquid chromatography (LC) system coupled with a quadrupole ion trap mass spectrometer (QTRAP^®^ 5500 LC-MS/MS System, AB Sciex, Framingham, MA, USA). Operating MS/MS conditions were the following: Nitrogen curtain gas flow rate of 25 L/min, collision gas glow rate of 9 L/min, ion spray source voltage of −4.5 kV, temperature of 350 °C, nebulizer gas flow rate of 35 L/min, and turbo gas flow rate of 30 L/min. Negative-mode of electrospray ionization (ESI^–^) was used. Qualification was based on multiple reaction monitoring (MRM) of selected ion pairs in the first quadrupole (Q1) and third quadrupole (Q3). The presence of compounds previously identified in aerial parts of amaranth [10,18,19,20,35] were verified.

### 2.11. Statistical Analysisl

The plant growth experiment was carried out in duplicate. Antioxidant assays and HPLC-DAD separations were performed for at least three repetitions. All results were presented as means ± standard deviations (SD). Significance of differences among mean values were estimated by one-way ANOVA and Fisher’s LSD test at a level of *p* < 0.05 (GraphPad Prism; GraphPad Software, San Diego, CA, USA). Principal component analysis (PCA) allowed examination of the relationships between TPC, individual phenolic compound contents and values of antioxidant assays obtained for amaranth at different growth stages.

## 3. Results and Discussion

### 3.1. Total Phenolic Content

The extraction yield and TPC of the aerial parts of amaranth at different growth stages, expressed on the basis of both extract and fresh matter, are reported in Table 1. The extraction yield did not differ statistically (*p* ≥ 0.05) for plants in vegetative, shooting and budding stages, while lower values were recorded for early flowering and grain fill stages of amaranth. The TPC ranged from 18.3 to 33.7 mg GAE/g extract. TPC expressed on the basis of plant fresh matter was in the range of 0.68 to 1.11 mg/g. The highest values were observed in the earliest (early and medium vegetative) and the oldest (early flowering and grain fill) stages of plant. The lowest TPC was noted for extracts obtained from the shooting and budding stages. Li et al. [19] compared phenolic contents of different botanical parts of three *Amaranthus* species (*A. hypochondriacus*, *A. caudatus*, and *A. cruentus*) and found that the leaves had the highest TPC while the seeds and stalks contained the lowest. Therefore, in our study, the high TPC levels found in the amaranth of vegetative stages could be related to the major proportion of leaves with respect to stalks present in these plant growth stages compared to the others. A similar trend was reported in other pseudocereals, such as quinoa [36], as well as soybean [32], where authors found that extracts obtained from the early and late vegetative stages were characterized by the highest TPC. The TPC of amaranth extract of early flowering stage plants presented in Table 1 was in agreement with those reported for methanol-water and acetone extracts from separate leaves (24.8–32.3 mg GAE/g extract) and flowers (27.2–33.3 mg GAE/g extract) collected from a flowering stage of *A. hybridus* [16]. In turn, higher TPC (2.9 mg GAE/g FM) was noted for the five-week-old *A. caudatus* leaves [26] corresponding to an intermediate between early and medium vegetative stages in our study.

### 3.2. Antioxidant Activity

**Figure 1:**
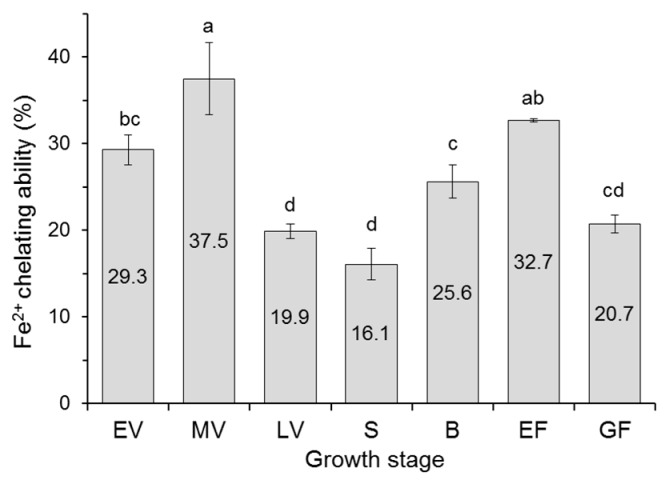
Fe^2+^ chelating ability of the amaranth extracts. EV, early vegetative; MV, medium vegetative; LV, late vegetative; S, shooting; B, budding; EF, early flowering; GF, grain fill. Different letters above bars indicate significant differences among means (*p* < 0.05).

**Figure 2:**
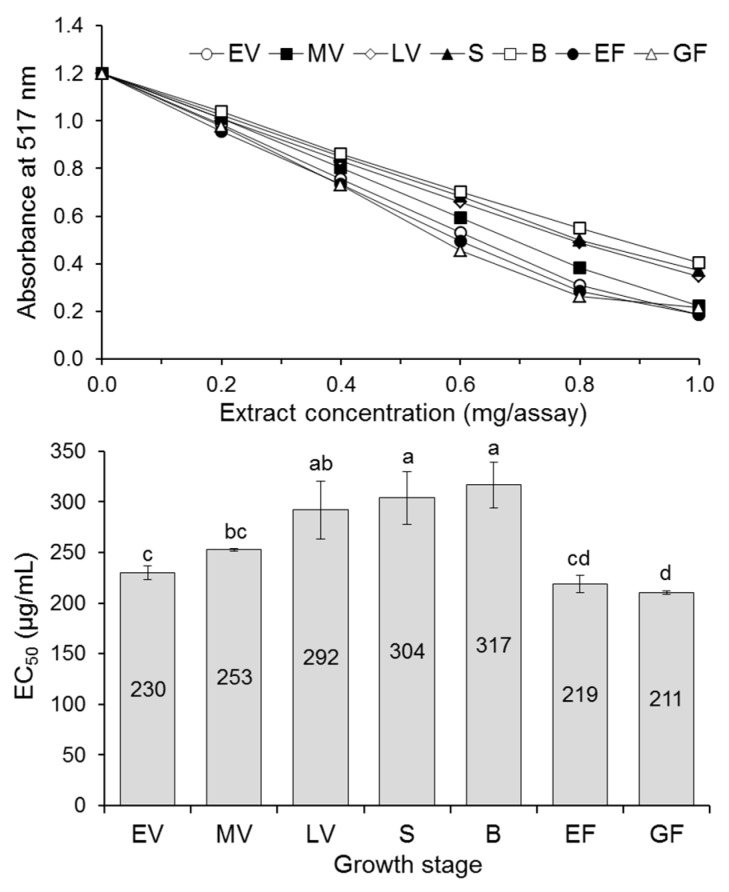
DPPH^•^ scavenging activity of the amaranth extracts. EV, early vegetative; MV, medium vegetative; LV, late vegetative; S, shooting; B, budding; EF, early flowering; GF, grain fill. Different letters above bars indicate significant differences among means (*p* < 0.05).

The antioxidant activity of amaranth samples of each growth stage was determined as radical scavenging activity (TEAC, PCL-ACL, and DPPH assay) and ability to reduce Fe^3+^ to Fe^2+^ (FRAP). Additionally, Fe^2+^ chelating ability of extracts was determined because phenolic compounds form stable complexes with ferrous ions, and in this way, decrease the extent of free ions to Fenton’s reaction in which highly reactive ^•^OH are generated [31]. The results of antioxidant activity are presented in Table 2 and Figure 1 and Figure 2. The significantly higher TEAC was recorded for amaranth in vegetative, early flowering and grain fill stages as opposed to in shooting and budding stages (*p* < 0.05), both when results were expressed on the basis of extract and fresh matter of plant. The FRAP ranged from 469 to 830 μmol Fe^2+^/g extract with the highest value observed for the early vegetative stage and the lowest, again, for the shooting and budding stages of the plant. The FRAP expressed on plant fresh matter basis was less varied between amaranth growth stages (17.4–24.6 μmol Fe^2+^/g). The PCL-ACL ranged from 422 to 858 μmol TE/g extract and from 16.5 to 23.9 μmol TE/g FM, respectively. For both types of PCL-ACL expression, amaranth in vegetative stages had significantly higher activity compared to plants in subsequent morphological states (*p* < 0.05). In turn, the Fe^2+^ chelating ability of extracts ranged from 16.1—19.9% for late vegetative and shooting stages to 37.5% for the medium vegetative stage (Figure 1). The changes of antiradical activity against DPPH^•^ of amaranth extracts with increasing assay content as well as the EC_50_ values, are presented in Figure 2. The DPPH^•^ scavenging activity expressed as EC_50_ showed significant differences between certain growth stages of amaranth (*p* < 0.05). The highest antiradical activity was obtained for extracts from plants in the early flowering and grain fill stages with the lowest EC_50_ value. The highest EC_50_ values (294–317 µg/mL), which did not differ statistically from each other (*p* ≥ 0.05), were found for late vegetative, shooting, and budding stages.

To the best of our knowledge, the changes of antioxidant activity of amaranth aerial parts during the growth cycle have not been previously demonstrated, although the differences in the antioxidant potential of various morphological parts of the plant were shown [16,19,37]. In general, FRAP, DPPH^•^, or ABTS^•+^ scavenging capacity decreased in this order: Leaves ≥ flowers >> stem > seeds. Among some amaranth species, this relationship was demonstrated for *A. caudatus* [19]. It is known that environmental factors, such as light and temperature, also play an important role in amaranth antioxidant metabolism. Khandaker et al. [38] found that the antioxidant activity of leaves of red amaranth (*A. tricolor*) was higher under full sunlight intensity than under dark conditions. Modi [39] studied the effect of growth temperature on yield, nutritional value, and antioxidant activity of the leaves of five *Amaranthus* spp. (*A. hybridus* var. *cruentus*, *A. hypochondriacus*, *A. tricolor*, *A. thunbergii*, and *A. hybridus*) harvested at 20, 40, and 60 days after sowing and found significant differences between growth temperature and stage of development. Authors concluded that for greater nutritional benefit, *Amaranthus* should be grown under warm conditions and that younger leaves are preferable. The maximum antioxidant activity, independent of the temperature and species considered, was found in leaves after 60 days of sowing, corresponding to an intermediate stage between late vegetative and shooting found in our study.

### 3.3. Phenolic Compound Profile

**Figure 3:**
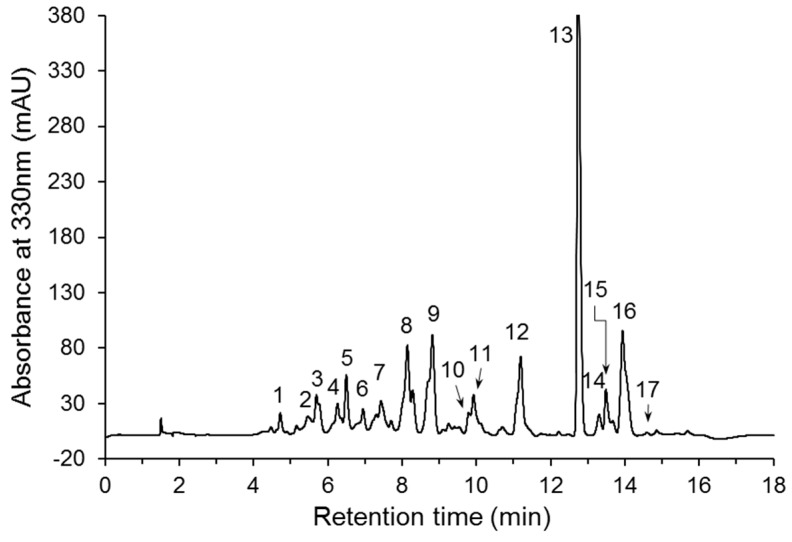
HPLC-DAD chromatogram of the phenolic compounds present in the amaranth extract.

The HPLC-DAD analysis showed that the phenolic profile was characterized by 17 compounds (Figure 3). The absorption maxima of UV-Vis spectra of compounds and MRM Q1/Q3 ion pairs used for their identification are listed in Table 3. Compounds **10**, **13**, and **17** were identified as caffeic acid, rutin, and kaempferol-3-*O*-rutinoside, respectively, by comparison with standards. Their presence in the extracts was confirmed by HPLC-MS/MS analysis. This analysis allowed also showing the presence of twelve hydroxycinnamic acids (1–12) and quercetin glucoside (15) in the extracts. Two other compounds (**14** and **16**) were tentatively identified as hydroxycinnamic acid derivatives based on the shape of UV spectra with maxima absorption at 315–329 nm and a shoulder at the shorter wavelength [34]. Most of the identified compounds (caffeic acid, caffeoyl-, coumaroyl- and feruloyl- glucaric isomers, feruloyl- and caffeoyl- quinic acids, rutin, kaempferol-3-*O*-rutinoside, and quercetin glucoside) were detected previously in leaves, seeds, and other aerial parts of *A. caudatus* [10,18,19,20]. Coumaroylquinic acids were found in stems of *A. spinosus* [35]. In turn, the free phenolic acids, such as, ferulic, *p*-coumaric, *p*-hydroxybenzoic, vanillic, sinapic, gallic, and protocatechuic acids, as well as betacyanins, were not identified in amaranth in the present study, although, according to literature data, they were determined in leaves, flowers, stalks and seeds of *A. caudatus* [10,11,19,40]. The lack of identification of betacyanins in our samples probably results from the selection of the betacyanin-free *A. caudatus* genotype for experiments.

The contents of individual phenolic compounds (**1**–**16**) in different growth stages of amaranth, expressed both per gram of extract and per gram of fresh matter of plant, are presented in Table 4 and Table 5, respectively. Compound **17** (kaempferol-3-*O*-rutinoside) was not quantified because its content in the samples was very low (< 1 µg/g FM). The predominant compound in all samples was rutin (15.0–36.2 mg/g extract; 418–1169 µg/g FM), and its content consisted of approximately 95% of the sum of flavonols. Compounds **11** and **16** were the most abundant hydroxycinnamic acid derivatives. The huge amount of rutin in comparison with other phenolic compounds was in line with literature data on aerial components, especially leaves, of different species of amaranth [19,20,41]. In turn, high content of hydroxycinnamic acid derivatives with very high quantity of caffeic acid derivatives was found by Neugart et al. [18] in *A. cruentus* leaves. These authors reported a 3.3–3.4-fold higher content of caffeic acid derivatives than quercetin glycosides in leaves harvested at the 8-12 leaf stage. In our study, the content of hydroxycinnamic acid derivatives predominated over the quantity of flavonols in the early vegetative stage, but with a lower difference between values (718 vs. 439 µg/g FM). Plant growth stage significantly affected the amount of individual phenolic compounds (Table 4 and Table 5). The rutin content (and sum of flavonols) increased for subsequent growth stages of plant, and the highest value was measured in early flowering and grain fill stages at the same level (*p* ≥ 0.05). Interestingly, on the contrary, the sum of hydroxycinnamic acid derivatives was found to be the highest in amaranth of the early vegetative stage, and this value decreased with age of amaranth for values expressed both on the basis of extract and fresh matter of plant. Our observation regarding the changes of rutin content during the amaranth growth cycle confirmed the trends previous reported by Kalinova and Dadakova [42]. These authors determined the rutin contents in leaves, flowers, stems, and seeds of six *Amaranthus* spp. (*A. caudatus*, *A. hypochondriacus*, *A. hybrid*, *A. retroflexus*, *A. cruentus*, and *A. tricolor*) and found that the rutin content in leaves was related to the developmental stage of the crop and that it usually increased with plant aging.

### 3.4. Principal Component Analysis

**Figure 4:**
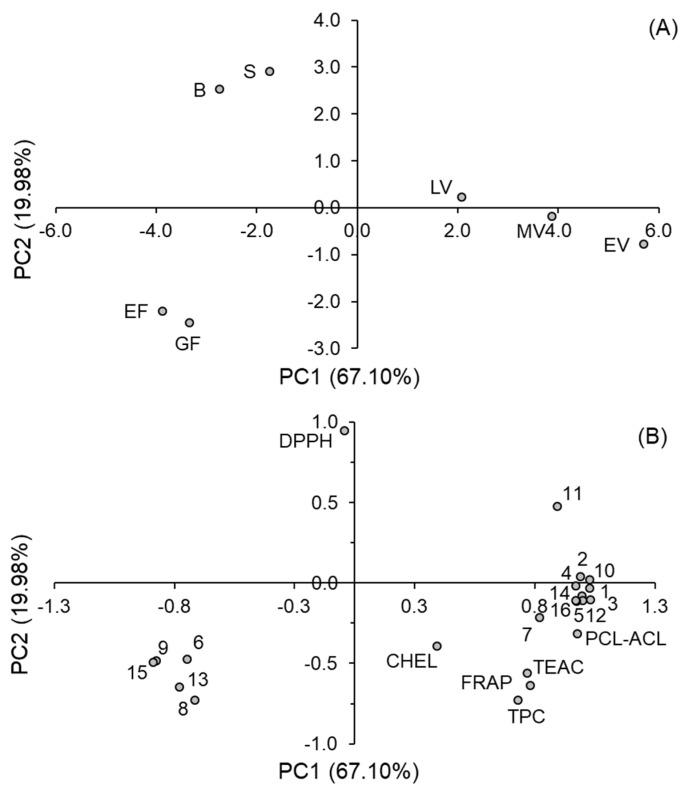
Principal component analysis (PCA) plots of the data set of variables: Total phenolic content (TPC), individual phenolic compound (**1**–**16**), and antioxidant assays obtained for amaranth in different growth stages. EV, early vegetative; MV, medium vegetative; LV, late vegetative; S, shooting; B, budding; EF, early flowering; GF, grain fill; TEAC, Trolox equivalent antioxidant capacity; FRAP, ferric-reducing antioxidant power; PCL-ACL, antioxidant capacity in photochemiluminescence assay; CHEL, Fe^2+^ chelating ability. (**A**): Distribution of growth stages, (**B**): Distribution of variables.

The PCA was carried out to clarify the relationship between the different growth stages of amaranth aerial parts and the evaluated variables. The data set obtained for 16 phenolic compounds, five antioxidant assays, and TPC with respect to seven growth stages was subjected to PCA. The results are presented in Figure 4. The first two principal components (PC1 and PC2) significantly explained most of the variations–87.08% of the total variance. The clustering of growth stages with three separate groups: 1–vegetative stages (early, medium, and late vegetative), 2–shooting and budding stages, and 3–early flowering and grain fill stages was obtained as shown in plot A (Figure 4). The variables that were differentiating distribution of each growth stage were depicted in plot B (Figure 4). The early, medium, and late vegetative stages were associated with the most antioxidant assays: TEAC, FRAP, PCL-ACL, and Fe^2+^ chelating ability. The TPC and content of some hydroxycinnamic acid derivatives (compounds **1**–**5**, **7**, **10**–**12**, **14**, and **16**) also affected this group of stages. In turn, the third group of growth stages (early flowering and grain fill) was clearly related to content of flavonols and three remaining hydroxycinnamic acid derivatives (compounds **6**, **8**, and **9**). The DPPH assay was negatively correlated with other antioxidant activity assays because the EC_50_ values were used in PCA. In the plot, the DPPH assay influenced the distribution of shooting and budding stages due to their low DPPH^•^ scavenging activity with high EC_50_ values.

## 4. Conclusions

Comparison of antioxidant activity and phenolic composition of the aerial parts of *A. caudatus* showed growth cycle dependent effects. The amaranth in shooting and budding stages had the lowest antioxidant activity and the lowest total phenolic content. The earlier (vegetative) and later (early flowering and grain fill) stages formed two groups, both with high antioxidant activity, though with differing dominant classes of phenolic compounds. The content of flavonols (mainly rutin) increased with growth cycle, and these compounds could be primarily responsible for the antioxidant activity of amaranth in early flowering and grain fill stages. In plants in vegetative stages, especially the early vegetative stage, the participation of hydroxycinnamic acid derivatives in the pool of phenolic compounds was high. The contribution of these compounds to high antioxidant activity of amaranth in vegetative stages seemed significant. It is worth noting the predominance of rutin quantity among individual phenolic compounds in all *A. caudatus* growth states, as well as its increasing content during the growth cycle.

The extracts obtained from amaranth in vegetative, early flowering, and grain fill stages, due to their high content of hydroxycinnamic acid derivatives and rutin, can be a valuable source of antioxidants that can be exploited for the production of nutraceuticals or used as a functional food ingredient.

## Figures and Tables

**Table 1:** Yield of extraction and total phenolic content (TPC) of the amaranth extract and fresh matter (FM) in different growth stages.

Growth Stage	Time after Seeding (days)	Extraction Yield (%)	TPC	
			mg GAE/g Extract	mg GAE/g FM
Early vegetative	34	24.3 ± 0.3 ^ab^	33.7 ± 4.5 ^a^	0.94 ± 0.14 ^abc^
Medium vegetative	41	26.3 ± 3.2 ^a^	31.1 ± 3.5 ^ab^	1.04 ± 0.05 ^ab^
Late vegetative	55	27.4 ± 0.6 ^a^	27.9 ± 0.4 ^b^	0.81 ± 0.03 ^bcd^
Shooting	62	26.7 ± 0.5 ^a^	18.6 ± 2.9 ^c^	0.68 ± 0.16 ^d^
Budding	69	26.6 ± 0.4 ^a^	18.3 ± 3.4 ^c^	0.71 ± 0.15 ^cd^
Early flowering	74	22.7 ± 0.5 ^bc^	27.4 ± 0.6 ^ab^	1.11 ± 0.03 ^a^
Grain fill	78	20.3 ± 1.7 ^c^	27.3 ± 3.5 ^ab^	0.88 ± 0.07 ^abc^

GAE, gallic acid equivalents. ^abcd^ Means with the different lowercase letters in the same column are significantly different (*p* < 0.05).

**Table 2:** Antioxidant activity of the amaranth extract and fresh matter (FM) in different growth stages.

Growth Stage	TEAC		FRAP		PCL-ACL	
	μmol TE/g Extract	μmol TE/g FM	μmol Fe^2+^/g Extract	μmol Fe^2+^/g FM	μmol TE/g Extract	μmol TE/g FM
Early vegetative	259 ± 27 ^ab^	7.23 ± 0.91 ^bc^	830 ± 27 ^a^	23.1 ± 1.3 ^ab^	858 ± 24 ^a^	23.9 ± 1.2 ^a^
Medium vegetative	283 ± 27 ^a^	9.52 ± 0.34 ^a^	665 ± 83 ^b^	22.3 ± 1.5 ^ab^	698 ± 43 ^b^	23.6 ± 2.8 ^a^
Late vegetative	272 ± 18 ^ab^	8.58 ± 0.38 ^ab^	648 ± 25 ^b^	20.4 ± 1.2 ^abc^	681 ± 42 ^b^	21.5 ± 1.8 ^ab^
Shooting	176 ± 6.0 ^c^	6.44 ± 0.69 ^c^	475 ± 31 ^c^	17.4 ± 2.4 ^c^	486 ± 3.9 ^cd^	17.8 ± 1.4 ^bc^
Budding	171 ± 8.1 ^c^	6.67 ± 0.53 ^c^	469 ± 75 ^c^	18.3 ± 3.5 ^bc^	422 ± 43 ^d^	16.5 ± 2.2 ^c^
Early flowering	218 ± 23 ^bc^	8.83 ± 0.87 ^ab^	606 ± 15 ^bc^	24.6 ± 0.4 ^a^	483 ± 20 ^cd^	19.6 ± 0.7 ^bc^
Grain fill	236 ± 45 ^ab^	7.62 ± 1.13 ^bc^	640 ± 112 ^b^	20.7 ± 2.7 ^abc^	558 ± 34 ^c^	18.1 ± 0.3 ^bc^

TEAC, Trolox equivalent (TE) antioxidant capacity; FRAP, ferric-reducing antioxidant power; PCL-ACL, photochemiluminescence-antioxidant capacity of lipid-soluble substances. ^abcd^ Means with the different lowercase letters in the same column are significantly different (*p* < 0.05).

**Table 3:** Chromatographic and spectral data of the phenolic compounds identified in amaranth extracts.

Compound No ^1^	t_R_ (min) ^2^	λ_max_ (nm) ^3^	Ion Pair ^4^ (Q1/Q3)	Compound
**1**	4.80	301sh;327	371/209; 371/173	Caffeoylglucaric acid 1
**2**	5.52	301sh;327	371/209; 371/173	Caffeoylglucaric acid 2
**3**	5.75	302sh; 327	371/209; 371/173	Caffeoylglucaric acid 3
**4**	6.31	302sh; 328	371/209; 371/173	Caffeoylglucaric acid 4
**5**	6.54	303sh; 328	371/209; 371/173	Caffeoylglucaric acid 5
**6**	6.95	304sh; 327	371/191; 371/209	Coumaroylglucaric acid 1
**7**	7.47	304sh; 327	371/191; 371/209	Coumaroylglucaric acid 2
**8**	8.18	299sh; 326	385/191; 385/209	Feruloylglucaric acid
**9**	8.84	303sh; 327	353/191; 353/173	Caffeoylquinic acid
**10**	9.78	296sh; 324	179/135; 179/107	Caffeic acid
**11**	9.93	301sh; 314	337/173; 337/155	Coumaroylquinic acid
**12**	11.21	304sh; 329	367/173; 367/155	Feruloylquinic acid
**13**	12.75	256, 354	609/301; 609/463	Rutin
**14**	13.32	302sh; 315	-	Hydroxycinnamic acid derivative
**15**	13.49	256, 354	463/301	Quercetin glucoside
**16**	13.95	303sh; 329	-	Hydroxycinnamic acid derivative
**17**	14.40	265; 348	593/285	Kaempferol-3-*O*-rutinoside

^1^ Compound number corresponds to peak number in Figure 3. ^2^ Retention time (t_R_) of HPLC-DAD separation. ^3^ Maximum absorption (λ_max_) of UV-Vis spectrum in HPLC-DAD analysis. ^4^ Ion pair of multiple reaction monitoring (MRM) of HPLC-MS/MS analysis.

**Table 4:** Individual phenolic compound contents in extracts of amaranth in different growth stages (mg/g).

Comp.No	Compound	Early Vegetative	Medium Vegetative	Late Vegetative	Shooting	Budding	Early Flowering	Grain Fill
**1** ^1^	Caffeoylglucaric acid 1	0.54 ± 0.08 ^a^	0.46 ± 0.09 ^a^	0.45 ± 0.01 ^a^	0.23 ± 0.01 ^b^	0.16 ± 0.01 ^b^	0.19 ± 0.06 ^b^	0.13 ± 0.03 ^b^
**2** ^1^	Caffeoylglucaric acid 2	0.77 ± 0.19 ^a^	0.91 ± 0.03 ^a^	0.83 ± 0.06 ^a^	0.43 ± 0.02 ^b^	0.35 ± 0.01 ^b^	0.26 ± 0.15 ^b^	0.41 ± 0.11 ^b^
**3** ^1^	Caffeoylglucaric acid 3	1.33 ± 0.15 ^ab^	1.60 ± 0.33 ^a^	1.19 ± 0.06 ^b^	0.59 ± 0.07 ^c^	0.47 ± 0.05 ^c^	0.52 ± 0.07 ^c^	0.47 ± 0.12 ^c^
**4** ^1^	Caffeoylglucaric acid 4	1.12 ± 0.22 ^a^	0.95 ± 0.37 ^ab^	1.13 ± 0.04 ^a^	0.60 ± 0.01 ^bc^	0.53 ± 0.01 ^c^	0.40 ± 0.01 ^c^	0.50 ± 0.12 ^c^
**5** ^1^	Caffeoylglucaric acid 5	1.70 ± 0.17 ^a^	1.25 ± 0.37 ^b^	1.20 ± 0.03 ^b^	0.60 ± 0.01 ^c^	0.50 ± 0.02 ^c^	0.53 ± 0.02 ^c^	0.47 ± 0.11 ^c^
**6** ^2^	Coumaroylglucaric acid 1	0.20 ± 0.03 ^bc^	0.11 ± 0.01 ^c^	0.23 ± 0.02 ^b^	0.20 ± 0.04 ^bc^	0.22 ± 0.06 ^b^	0.39 ± 0.07 ^a^	0.08 ± 0.05 ^b^
**7** ^2^	Coumaroylglucaric acid 2	0.61 ± 0.14 ^b^	0.72 ± 0.09 ^a^	0.63 ± 0.01 ^ab^	0.44 ± 0.02 ^bc^	0.39 ± 0.05 ^c^	0.34 ± 0.00 ^c^	0.58 ± 0.14 ^ab^
**8** ^3^	Feruloylglucaric acid	1.00 ± 0.07 ^b^	1.16 ± 0.47 ^ab^	1.21 ± 0.08 ^ab^	1.04 ± 0.09 ^b^	1.17 ± 0.21 ^ab^	1.67 ± 0.04 ^a^	1.74 ± 0.39 ^a^
**9** ^1^	Caffeoylquinic acid	0.79 ± 0.10 ^c^	0.73 ± 0.10 ^c^	1.08 ± 0.14 ^bc^	0.97 ± 0.15 ^bc^	1.11 ± 0.21 ^bc^	1.60 ± 0.19 ^a^	1.42 ± 0.36 ^ab^
**10**	Caffeic acid	0.84 ± 0.03 ^a^	0.69 ± 0.22 ^b^	0.39 ± 0.02 ^c^	0.20 ± 0.01 ^cd^	0.12 ± 0.03 ^d^	0.09 ± 0.02^d^	Tr
**11** ^2^	Coumaroylquinic acid	0.63 ±0.09 ^a^	0.59 ± 0.13 ^a^	0.57 ± 0.10 ^a^	0.53 ± 0.05 ^a^	0.44 ± 0.06 ^ab^	0.33 ±0.04 ^bc^	0.22 ± 0.03 ^c^
**12** ^3^	Feruloylquinic acid	9.89 ± 0.07 ^a^	5.17 ± 1.11 ^b^	3.54 ± 0.09 ^c^	1.27 ± 0.03 ^d^	0.46 ± 0.18 ^e^	0.39 ± 0.01 ^e^	0.34 ± 0.01 ^e^
**13**	Rutin	15.0 ± 1.8 ^b^	19.7 ± 5.7 ^b^	19.3 ± 4.5 ^b^	19.6 ± 2.8 ^b^	20 ± 2.7 ^b^	31.7 ± 2.7 ^a^	36.2 ± 8.2 ^a^
**14** ^2^	Hydroxycinnamic acid derivative	1.55 ± 0.01 ^a^	0.69 ± 0.17 ^b^	0.59 ± 0.10 ^b^	0.17 ± 0.1 ^c^	0.23 ± 0.05 ^c^	0.09 ± 0.01 ^c^	0.18 ± 0.00 ^c^
**15** ^4^	Quercetin glucoside	0.78 ± 0.07 ^c^	0.86 ± 0.21 ^c^	1.34 ± 0.57 ^abc^	1.23 ± 0.15 ^bc^	1.28 ± 0.03 ^bc^	1.97 ± 0.16 ^ab^	2.07 ± 0.60 ^a^
**16** ^2^	Hydroxycinnamic acid derivative	4.80 ± 0.10 ^a^	2.51 ± 0.70 ^b^	2.83 ± 0.49 ^b^	1.59 ± 0.23 ^c^	1.11 ± 0.27 ^c^	1.26 ± 0.17 ^c^	1.17 ± 0.08 ^c^
**17**	Kaempferol-3-*O*-rutinoside	Tr	Tr	Tr	Tr	Tr	Tr	Tr
Sum of all compounds		41.5 ± 2.8 ^a^	38.1 ± 2.7 ^abc^	36.5 ± 6.3 ^abc^	29.6 ± 3.3 ^bc^	28.5 ± 3.7 ^c^	41.8 ± 2.8 ^ab^	46.1 ± 9.0 ^a^
Sum of hydroxycinnamic acids		25.8 ± 1.0 ^a^	17.5 ± 2.8 ^b^	15.9 ± 1.2 ^b^	8.86 ± 0.38 ^c^	7.27 ± 1.03 ^c^	7.99 ± 0.03 ^c^	7.91 ± 1.50 ^c^
Sum of flavonols		15.79 ± 1.8 ^b^	20.6 ± 6.0 ^b^	20.6 ± 5.1 ^b^	20.8 ± 2.9 ^b^	21.2 ± 2.7 ^b^	33.7 ± 2.8 ^a^	38.2 ± 8.8 ^a^

^1^ Expressed as caffeic acid equivalents. ^2^ Expressed as *p*-coumaric acid equivalents. ^3^ Expressed as ferulic acid equivalents. ^4^ Expressed as quercetin equivalents. Tr, traces. ^abcd^ Means with different lowercase letters in the same row are significantly different (*p* < 0.05). Compound number corresponds to peak number in Figure 3.

**Table 5:** Individual phenolic compound contents in fresh matter of amaranth in different growth stages (µg/g).

Comp.No	Compound	Early Vegetative	Medium Vegetative	Late Vegetative	Shooting	Budding	Early Flowering	Grain Fill
**1** ^1^	Caffeoylglucaric acid 1	14.9 ± 1.8 ^a^	15.4 ± 4.0 ^a^	14.1 ± 0.1 ^a^	8.36 ± 0.98 ^b^	6.36 ± 0.54 ^b^	7.63 ± 2.36 ^b^	4.16 ± 0.60 ^b^
**2** ^1^	Caffeoylglucaric acid 2	21.3 ± 4.7 ^bc^	30.6 ± 0.9 ^a^	26.1 ± 1.3 ^ab^	15.6 ± 0.3 ^cd^	13.7 ± 0.7 ^cd^	10.6 ± 6.4 ^d^	13.3 ± 3.0 ^d^
**3** ^1^	Caffeoylglucaric acid 3	37.1 ± 3.4 ^b^	53.4 ± 7.9 ^a^	37.7 ± 1.0 ^b^	21.6 ± 2.5 ^c^	18.2 ± 2.4 ^c^	21.1 ± 3.0 ^c^	15.1 ± 3.3 ^c^
**4** ^1^	Caffeoylglucaric acid 4	31.1 ± 5.5 ^ab^	32.2 ± 14 ^ab^	35.7 ± 0.5 ^a^	21.9 ± 1.8 ^abc^	20.6 ± 1.2 ^bc^	16.4 ± 0.3 ^c^	16.0 ± 3.3 ^c^
**5** ^1^	Caffeoylglucaric acid 5	47.3 ± 3.6 ^a^	42.5 ± 11 ^a^	37.7 ± 0.1 ^a^	21.8 ± 2.0 ^b^	19.7 ± 1.2 ^b^	21.6 ± 0.9 ^b^	15.2 ± 2.9 ^b^
**6** ^2^	Coumaroylglucaric acid 1	5.46 ± 0.07 ^bc^	3.59 ± 0.04 ^c^	7.22 ± 0.06 ^bc^	7.51 ± 0.9 ^bc^	8.81 ± 2.5 ^b^	16.0 ± 3.2 ^a^	9.07 ± 1.24 ^b^
**7** ^2^	Coumaroylglucaric acid 2	16.8 ± 3.6 ^bc^	24.3 ± 1.6 ^a^	19.9 ± 0.0 ^ab^	16.0 ± 2.0 ^bc^	15.3 ± 2.4 ^bc^	13.7 ± 0.0 ^c^	18.6 ± 3.7 ^bc^
**8** ^3^	Feruloylglucaric acid	27.9 ± 1.2 ^c^	39.4 ± 15.2 ^bc^	38.1 ± 1.7 ^bc^	38.0 ± 6.1 ^bc^	45.9 ± 9.5 ^bc^	67.7 ± 1.3 ^a^	56.1 ± 0.10 ^ab^
**9** ^1^	Caffeoylquinic acid	22.0 ± 2.3 ^c^	24.8 ± 4.7 ^c^	34.1 ± 3.7 ^bc^	35.7 ± 8.0 ^bc^	43.3 ± 9.5 ^b^	65.0 ± 7.2 ^a^	45.9 ± 9.6 ^b^
**10**	Caffeic acid	26.2 ± 0.3 ^a^	22.7 ± 5.8 ^a^	12.2 ± 0.4 ^b^	7.24 ± 0.74 ^bc^	4.69 ± 1.21 ^c^	3.04 ± 0.09 ^c^	Tr
**11** ^2^	Coumaroylquinic acid	17.6 ± 3.1 ^a^	20.2 ± 5.7 ^a^	17.9 ± 2.8 ^a^	19.6 ± 3.2 ^a^	17.1 ± 1.7 ^a^	13.4 ± 1.8 ^ab^	7.28 ± 0.8 ^bc^
**12** ^3^	Feruloylquinic acid	275 ± 4.2 ^a^	175 ± 48 ^b^	111 ± 1.0 ^c^	46.2 ± 2.2 ^d^	18.1 ± 7.5 ^d^	15.8 ± 0.4 ^d^	11.2 ± 0.3 ^d^
**13**	Rutin	418 ± 40 ^c^	658 ± 155 ^bc^	606 ± 129 ^bc^	718 ± 154 ^bc^	780 ± 130 ^b^	1286 ± 99 ^a^	1169 ± 215 ^a^
**14** ^2^	Hydroxycinnamic acid derivative	40.6 ± 0.7 ^a^	23.3 ± 7.2 ^b^	18.4 ± 2.8 ^b^	6.11 ± 3.44 ^c^	9.00 ± 2.25 ^c^	3.55 ± 0.48 ^c^	5.88 ± 0.30 ^c^
**15** ^4^	Quercetin glucoside	21.6 ± 2.5 ^c^	29.3 ± 8.7 ^c^	41.9 ± 17 ^bc^	45.1 ± 8.6 ^bc^	50.0 ± 2.9 ^b^	79.6 ± 5.8 ^a^	66.7 ± 0.17 ^ab^
**16** ^2^	Hydroxycinnamic acid derivative	134 ± 5.7 ^a^	85.1 ± 28.7 ^b^	89.0 ± 13.8 ^b^	58.5 ± 12.7 ^bc^	43.4 ± 9.8 ^c^	51.1 ± 6.5 ^c^	38.0 ± 0.8 ^c^
**17**	Kaempferol-3-*O*-rutinoside	Tr	Tr	Tr	Tr	Tr	Tr	Tr
Sum of all compounds		1157 ± 51 ^bc^	1281 ± 14 ^bc^	1147 ± 175 ^bc^	1087 ± 196 ^c^	1114 ± 181 ^c^	1690 ± 101 ^a^	1491 ± 270 ^ab^
Sum of hydroxycinnamic acids		718 ± 14 ^a^	593 ± 132 ^ab^	499 ± 29 ^b^	324 ± 38 ^c^	264 ± 49 ^c^	324 ± 3.9 ^c^	256 ± 39 ^c^
Sum of flavonols		439 ± 42 ^c^	688 ± 163 ^bc^	648 ± 146 ^bc^	763 ± 163 ^bc^	830 ± 132 ^b^	1366 ± 105 ^a^	1235 ± 232 ^a^

^1^ Expressed as caffeic acid equivalents. ^2^ Expressed as *p*-coumaric acid equivalents. ^3^ Expressed as ferulic acid equivalents. ^4^ Expressed as quercetin equivalents. Tr, traces. ^abcd^ Means with different lowercase letters in the same row are significantly different (*p* < 0.05). Compound number corresponds to peak number in Figure 3.
